# First Molecular Detection of *Toxoplasma gondii* DNA in Blood and Milk of Goats from Algeria

**DOI:** 10.3390/pathogens14020174

**Published:** 2025-02-10

**Authors:** Abdeldjalil Dahmane, Alice Vismarra, Karine Passebosc-Faure, Nassiba Reghaissia, Djamel Baroudi, Houssem Samari, Manuela Semeraro, Hélène Yera, AbdElkarim Laatamna

**Affiliations:** 1 Food Hygiene and Quality Assurance System (HASAQ) Research Laboratory, Department of Veterinary Sciences, Higher National Veterinary School, Issad Abbas Street, Bab Ezzouar, 16000 Algiers, Algeria; a.dahmane@etud.ensv.dz (A.D.); dbaroudi7@hotmail.com (D.B.); 2 Laboratory of Exploration and Valorization of Steppic Ecosystems, Faculty of Nature and Life Sciences, University of Djelfa, Moudjbara Road, 17000 Djelfa, Algeria; 3 Parasitology and Parasitic Diseases Unit, Department of Veterinary and Medical Sciences, University of Parma, Via del Taglio 10, 43126 Parma, Italy; manuela.semeraro@unipr.it; 4 Centre National de Référence (CNR) Toxoplasmose, Centre Hospitalier-Universitaire Limoges, Inserm U1094, IRD UMR270, Université de Limoges, EpiMaCT, 87042 Limoges, France; karine.passebosc-faure@chu-limoges.fr (K.P.-F.); helene.yera@chu-limoges.fr (H.Y.); 5 Institute of Veterinary and Agronomic Sciences, University of Souk Ahras, Annaba Road, 41000 Souk Ahras, Algeria; n.reghaissia@univ-soukahras.dz; 6 Faculty of Sciences, University of M’sila, 28000 M’sila, Algeria; houssem.samari@univ-msila.dz

**Keywords:** toxoplasmosis, ELISA, PCR, MS genotyping, goats, one health

## Abstract

Toxoplasmosis is one of the most important foodborne diseases in humans, potentially acquired by ingesting unpasteurized goat milk. This study examined the role of goat milk as a source of infection of *Toxoplasma gondii* for humans in Algeria. Sera, blood, and milk samples collected from 106 female goats were tested for the presence of antibodies against *T. gondii* and its DNA, using indirect ELISA and PCR, respectively. Multiplex PCR was performed using 15 microsatellite markers to determine the clonal type of the *T. gondii* DNA detected. Seropositive results were found in 51 she-goats (48.11%). *T. gondii* DNA was detected in 16 (15.09%) and 15 (14.15%) blood and milk samples, respectively. In total, 15 (29.41%) out of 51-seropositive goats were PCR-positive for blood, while only 6 of them (6/15, 40%) showed the presence of *T. gondii* DNA in their milk. A fair correlation was found between indirect ELISA and PCR assays for *T. gondii* detection in milk (K = 0.2243) and blood (K = 0.28300), with a substantial difference in the screening ability of the tests (G2 = 38.96, *p* < 0.0001). The genotyping of samples could not be completed, but showed the absence of type I and type III lineages in goats from the Mila region, northeastern Algeria. The Algerian goat population is highly exposed to *T. gondii*, with a potentially increased risk of parasite transmission to humans via milk consumption.

## 1. Introduction

*Toxoplasma gondii*, a protozoan parasite from the phylum Apicomplexa, is the causative agent of toxoplasmosis, a widespread zoonotic disease. Warm-blooded animal species, including humans, are frequently infected with *T. gondii*, and felines are the only definitive hosts of the parasite [1].

According to Dubey [2], up to one-third of the world’s population is chronically infected. Human infections are often asymptomatic, however, certain patients, particularly immunocompromised individuals (e.g., AIDS patients) and transplant recipients, may experience lymphadenopathy, ocular toxoplasmosis, and even fatal infections [3,4]. *T. gondii* infection in pregnant women may result in abortion, stillbirth, or other significant effects in newborns, especially ocular disease [3,5].

The parasite may spread between distinct definitive hosts or between different intermediate hosts, as well as from a definitive host to an intermediate host and vice versa [5]. Sheep and goats are commonly acknowledged as one of the principal animal food sources for human infection [2], and this foodborne disease represents a significant burden in several nations [6,7]. Human infection is mainly acquired via the consumption of undercooked meat (of several animal species, including sheep), through the consumption of water and vegetables contaminated with sporulated oocysts, and also through the consumption of unpasteurized milk from infected goats [2,8].

Indeed, *T. gondii* tachyzoites have been identified in milk from different animal species, including cows, goats, and sheep [5,9]. *T. gondii* DNA has been found in cheese made from the unpasteurized and raw milk of both experimentally and naturally infected goats [10,11,12]. Several studies have linked unpasteurized goat milk consumption to symptomatic and even fatal toxoplasmosis infections in humans [13,14]. Iacobucci et al. [15] suggested that there are conflicting data concerning the possibility of human infection with *T. gondii* via the ingestion of unpasteurized milk. However, *T. gondii* has been demonstrated to be viable in goat milk and to exist in sufficient quantities in both raw milk and cheese products to infect mice and cats [12]. Limited studies have confirmed the presence of *T. gondii* in goat milk either through cat bioassay and/or PCR, with values ranging between 6 and 9.4% [16,17]. In North Africa, data about *T. gondii* detection in milk and dairy products from goats are scarce.

According to Ghoneim et al. [18], 41.7% and 25% of goats in Egypt had positive results using ELISA and PCR, respectively. In a Moroccan serological survey, 8.5% (9/106) of female goats tested positive for toxoplasmosis [19]. Seroprevalence in female goats was recently determined to be 31.2% in Tunisia [20].

Serological screening in goats from Algeria showed prevalence values ranging from 11.92% to 71.74%, with an average prevalence of 33.61% [21]. According to research on the epidemiology of toxoplasmosis in goats conducted in the Mila area of northeastern Algeria, *T. gondii* infection has a high seroprevalence rate of 63.58% [22]. In a recent study, Dahmane et al. [23] revealed a high prevalence rate of 53.26% and a level infection of 51.85% in the Mila region, where the current study was conducted. These findings support the idea that the human consumption of milk from this species might result in infection.

In some ethnic and religious groups, milk and traditional milk products are revered and are of great dietary significance [15]. There is little information available on the transmission of toxoplasmosis in Algeria through the intake of meat and dairy products, despite the population’s reliance on livestock and the close connection between herding families’ health and that of their animals. Moreover, the absence of studies involving caprine milk as a potential source of toxoplasmosis transmission in Algeria highlights the importance of conducting the current study, also because the seroprevalence reported in pregnant women is still quite high (51.6%) [24,25].

The present study aimed to determine (a) the seroprevalence of *T. gondii* infection in female goats, (b) the occurrence of *T. gondii* DNA in blood and milk samples, providing a preliminary assessment of contamination risk with *T. gondii* in Algerian goat milk, and (c) the molecular characterization of *T. gondii* using multiplex PCR targeting 15 microsatellite markers, and simplex PCR if necessary.

## 2. Materials and Methods

### 2.1. Study Area and Environment

**Figure 1:**
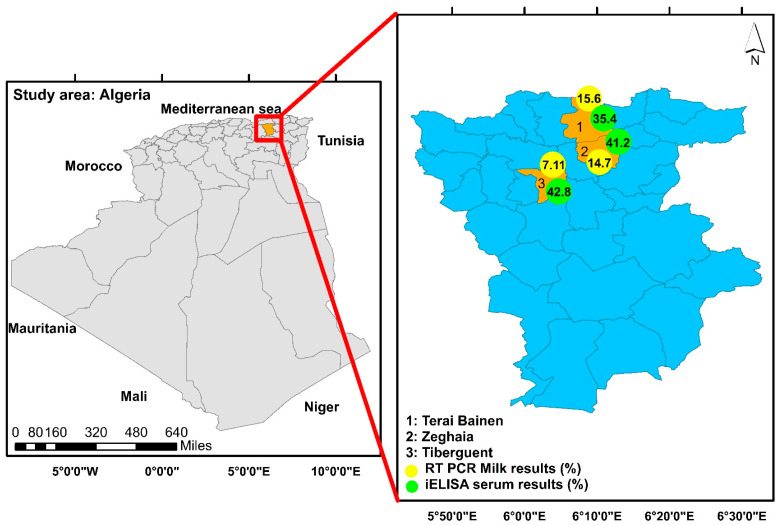
Map of the study area and prevalence rates *T. gondii* in goats from Mila, northeastern Algeria.

The present study was approved by the scientific committee of the Higher National Veterinary School, Algiers (code number 270/DPGR/2018). It was carried out in Mila province, which is located in northeastern Algeria (Figure 1).

Mila lies inland, about 82 km from the Mediterranean coast. The district is characterized by varied relief and presents the following two large distinct zones: to the north, the mountains, and the south, the plains and highlands, with an area of 3481 km^2^. The region has a humid Mediterranean climate, with hot, dry summers and cold, wet winters. Its annual rainfall is approximately 550 mm with a relative humidity of 70%, and the annual temperature varies between 8 °C and 24 °C [26]. Livestock husbandry in this province consists of a mixture of breeding cattle and small ruminants. According to data from recent years (2019–2020) from the Algerian Ministry of Agriculture, approximatively 35,136 goat heads are raised in Mila [27].

### 2.2. Target Population and Sampling

The sample size was calculated by the statistical formula presented by Thrusfield [28], with an expected molecular prevalence of 7% [20], an expected error of 5%, and assuming a 95% confidence interval, as follows:

N = [Z2 × P (1 − P)]/d2

where:

- N is the number of samples to be collected in the study;
- Z is the value of the normal distribution for the confidence interval of 95% [Z = 1.96];
- P is the expected prevalence;
- d is the absolute error or required precision of ±5% for a 95% confidence interval (0.05).

A minimum of 100 healthy female goats was required. Thirty-one dairy herds were randomly selected and the herd sizes ranged from five to fifteen heads.

At the individual level, the sample size was determined for each flock for the eventual detection of *T. gondii* DNA. The calculations were performed according to the formula commonly used in veterinary epidemiological surveys [28], as follows:

n = ([1 − (1 − p) 1/d] × [N − (d/2)]) + 1

where:

n is the size of the sample in each flock,

p is the probability of the detection of at least one positive goat in a herd determined at 95%,

N is the size of the flock,

d is the number of positive goats in the herd (this was calculated assuming that within-herd prevalence equals 10%).

The number of selected goats per herd varied from 2 to 6 depending on the total number and permission of the animal owner for sample collection (Table S1).

A total of 318 biological samples of blood, sera, and raw milk were taken from 106 clinically healthy female goats that were aged from one to eight years old. The samples were collected in 2020 and 2021 from subsistence properties in the province of Mila, northeastern Algeria. These samples were collected from 31 dairy farms. The properties were chosen based on convenience, depending on ease of access.

Blood samples (2–3 mL) were collected from each animal by venipuncture of the jugular with sterile 40 × 12 cm needles in a vacuum tube (Vacutainer^®^), without anticoagulant, and they were centrifuged at 2000× *g* for 10 min to completely extract the sera, which were stored at −20 °C until serological testing. In addition, 2–3 mL of blood was also collected into a tube containing EDTA and stored at −20 °C until molecular investigation.

Raw milk was obtained from lactating goats (at the first (1–45 days), the second (46–90 days), and the third (91–120 days) phase of lactation) by manually milking the teats through previous disinfection with an alcohol solution (70°) and the use of gloves during manipulation. During milk collection, the first few squirts were ignored. The samples were individually stored in sterile polypropylene V-shaped-bottom tubes DNAase- and RNAase-free, at a final volume of 10 mL, and kept under refrigeration until arrival at the laboratory, where they were stored at −20 °C until the implementation of molecular analysis.

A questionnaire was distributed to collect the epidemiological data from the visited farms (age, presence of cats, history of abortion, and management system) (see Table S1).

### 2.3. Serological Analysis

All sera samples (*n* = 106) were tested for *T. gondii* IgG antibody detection using the ID Screen Toxoplasmosis Indirect ELISA Multi-species kit (ID Screen, ID.VET Innovative Diagnostics, Montpellier, France), according to the manufacturer’s instructions. This is an indirect ELISA (iELISA) that uses the native P30 (SAG1) antigen and the anti-multi-species conjugate as the secondary antibody. This configuration is effective for identifying *T. gondii*-specific antibodies present in the serum of ruminants, pigs, dogs, and cats, as well as in milk and meat juice [29]. The results are expressed as a percentage of the optical density (OD) reading of the test, calculated as %OD = 100 × (OD sample − OD Negative Control)/(OD Positive Control—OD Negative Control). The samples were considered to be positive if they had a value of ≥50%, doubtful for values between 40% and 50%, and negative with a value of ≤40%.

### 2.4. DNA Extraction and T. gondii PCR Reactions

Blood and milk samples from the goats were processed for DNA extraction and subsequent amplification by PCR. DNA was extracted from both types of biological samples (blood and milk) using a ReliaPrep™ Blood gDNA Miniprep System kit (Promega, Madison, WI, USA), in accordance with the manufacturer’s instructions. Before DNA extraction, pretreatment of the milk samples was performed, as described by Mancianti et al. [11]. Briefly, the milk samples were centrifuged at 2200× *g* for 5 min. To avoid interference with casein, 1 mL of the pellet was treated with 200 µL of TE (1 mM EDTA, 10 mM Tris-HCl (pH = 7.6)) and 300 µL 0.5 M EDTA (pH = 8). The solution was then centrifuged at 3000× *g* for 10 min and finally diluted in 200 µL of PBS. After extraction, the DNA was stored in Eppendorf tubes at −20 °C until use.

DNA was then analyzed with a PCR protocol targeting the REP 529 bp marker of *T. gondii*. It was reported that REP 529 bp amplification through PCR showed the best *T. gondii* detection results among other methods targeting other genes, such as the B1 gene. REP 529 PCR assays can detect as few as 1–10 tachyzoites in clinical samples such as blood and milk [30]. Positive controls (DNA of *T. gondii* tachyzoites extracted from reference RH strains cultured in the laboratory) and negative controls were included in all reactions. This protocol was a real-time PCR (RT-PCR) described by Edvinsson et al. [30], and the primers used were TOX4 (CGCTGCAGGGAGGAAGACGAAAGTTG) and TOX5 (C GCTGCAGACACAGTGCATCTGGATT). The amplification protocol was characterized by a denaturation step (95 °C for 5 min) and 45 repeated cycles (95 °C—15 s; 58.5 °C—30 s). Fluorescence signals were collected in every cycle, and the presence of a specific product was avoided through a melting curve analysis (where *T. gondii* has a melting temperature of 82.5–83 °C). Positive samples with a significant amount of parasitic DNA (ct < 32) were sent for genotyping in the Epidemiology Strains Pole of the French Reference Center for Toxoplasmosis (CNR Toxoplasmose, Limoges site).

### 2.5. Genotyping of T. gondii DNA

Four samples, three that were extracted from milk and one from blood, resulted in being positive with RT-PCR designed for genotyping based on 15 microsatellite (MS) markers distributed across 11 of the 13 chromosomes composing the *T. gondii* genome in a single multiplex PCR assay, as described previously [31]. These 15 loci included a combination of 8 typing markers with low polymorphism (TUB2, W35, TgM-A, B17, B18, M33, IV.1, and XI.1) that showed little or no variation within lineages and 7 fingerprinting markers (M48, M102, N83, N82, AA, N61, and N60) exhibiting high polymorphism and a significant variation within lineages. We also genotyped all positive controls (RH, ME49, and VEG) using the same MS typing. As the amount of *T. gondii* DNA was insufficient for 15MS typing, we aimed to amplify some of the main typing markers in fourplex (with four different MS markers) or in simplex PCR reactions to differentiate as best as possible the possible strain lineage. We used the same protocol as the 15MS PCR. PCR products were sized using capillary electrophoresis on ABI PRISM 3130xl (Applied Biosystems, Foster City, CA, USA) and the GenScan 500 ROX dye size standard (Applied Biosystems). The results were analyzed using the GeneMapper 5.0 software packages (Applied Biosystems).

### 2.6. Statistical Analysis

The results of the biomolecular and serological tests were recorded in an Excel^®^ spreadsheet (version 2016, Microsoft Corporation, Redmond, WA, USA) and descriptive statistics were performed to calculate the frequencies and percentages of the results for the serum, blood, and milk samples. The significance of differences in the prevalence rates and risk factor assessment was analyzed with chi-square test (two-tailed) and odds ratios at 95% confidence intervals using an online statistical software, www.vassarstats.net (accessed dates; 24–26 October 2023), as described previously [32]. *p*-values and odds ratio were also confirmed with SPSS version 22.0 (IBM SPSS Inc., Chicago, IL, USA). The results were considered to be significant when the *p*-value was <0.05.

To verify if the presence of *T. gondii* antibodies in sera and its DNA in milk could be influenced by any of the considered variables, the presence/absence of IgG antibodies and the parasite DNA in serum/milk were used as the response variables, and the following independent variables were entered in the model: locality, age, phase of lactation, management system, abortion history, presence of cats, and season. Variables with a *p*-value of <0.05 in a univariate analysis were offered to the multivariate analysis, if it was applicable for at least two factors.

A Log-linear statistical analysis (a version of chi-square analysis) was performed to show the relationship between the serological and molecular results in serum–blood–milk triplet samples. To confirm the correspondence between the sera and milk results, a Two-Tailed McNemar Test was computed. The concordance between the PCR and ELISA results was estimated with the calculation of the Linear Weighted Kappa Coefficient; kappa values from 0.00 to 0.20 were considered as light, 0.21 to 0.40 as fair, 0.41 to 0.60 as moderate, 0.61 to 0.80 as substantial, and 0.81 to 1.00 as almost perfect [33].

## 3. Results

### 3.1. Serological Prevalence

Seropositive goats were found on all farms (100%). Overall, 51 out of 106 (48.11%) female goat serum samples scored positive for anti-*T. gondii* IgG using indirect ELISA.

### 3.2. Molecular Detection in Blood and Milk

Among the 106 goats, the RT-PCR assay gave positive results for blood samples from 16 of them (15.09%) and for 15 of milk samples (14.15%). The kappa coefficient for the association between the results of the RT-PCR for blood and milk samples was 0.35780, with a fair level of agreement (Table 1), indicating that approximately one half (7/16, 43.75%) of goats with circulating parasite DNA were also excreting parasite DNA in their milk, and 8/15 (53.33%) of goats were excreting the parasite DNA in their milk without presenting circulating parasite DNA. The McNemar test indicated a statistically non-significant difference between the rates of *T. gondii* DNA in blood and milk (Table 1).

Among the seropositive goats (*n* = 51), *T. gondii* DNA was detected in 15 blood samples (29.41%) and 13 milk samples (25.49%) using RT-PCR assay. The kappa coefficient for the association between the results of the RT-PCR was 0.2139, indicating a fair level of agreement (Table 2), and the analysis showed that all of these fifteen seropositive goats had positive RT-PCR results for blood, but only six of them (6/15, 40%) were excreting *T. gondii* DNA in their milk Statistically, based on the McNemar test, a non-significant association was found between the proportions of goats with anti-*T. gondii* antibodies and a positive RT-PCR in both their blood and milk samples (Table 2).

### 3.3. Comparison Between iELISA and RT-PCR in Milk

Based on the serological and molecular results, statistical analysis via the McNemar test found that the prevalence rates of *T. gondii* infection were strongly significantly different in sera and milk samples (*p* < 0.001) (Table 3). Therefore, an indirect ELISA test seemed to be able to detect a previous exposure to the parasite 19 times more than RT-PCR assay applied to milk samples. The kappa coefficient between the two tests was low (K = 0.2243), reflecting a fair level of agreement (Table 3). The analysis of milk by RT-PCR indicated that 40 serum–milk pairs were not in accordance. Twenty-three positive serum samples had negative corresponding milk samples, while two negative serum samples had positive corresponding milk samples.

Regarding molecular detection in milk using RT-PCR assay, *T. gondii* DNA was amplified in 15 out of 106 tested samples. The results for the presence of *T. gondii* DNA in milk samples from seropositive goats were considered. *T. gondii* DNA was only detected in 13 milk samples out of 51 (20.6%) seropositive animals. Only two seronegative goats showed RT-PCR positivity in their milk, while the other goats resulting seronegative did not show *T. gondii* DNA in their milk at the sampling time (Table 3).

### 3.4. Comparison Between iELISA and RT-PCR in Blood

Based on the McNemar test analysis, a highly significant difference was recorded between the prevalence rates of *T. gondii* infection in the serum and blood samples (*p* < 0.000001) (Table 4). Moreover, the indirect ELISA used in the sera appeared to be suitable for detecting the parasite 36 times more than the RT-PCR assay applied to the blood samples, with a limited correlation (Table 4).

A total number of 16 blood samples were RT-PCR-positive (15.09%). Among seropositive goats (*n* = 51), 15 blood samples were positive in RT-PCR (29.41%). In addition, only one seronegative goat had parasite DNA in its corresponding blood sample (1/55, 1.81%). A fair level of agreement was found between iELISA and RT-PCR (K = 0.2830) (Table 4).

### 3.5. Comparison Between Results of iELISA and RT-PCR in Blood and Milk

While anti-*T. gondii* antibodies were detected in 51/106 (48.11%) of the examined goat serum samples, the samples were subjected to further examination using RT-PCR for the detection of *T. gondii* DNA, which was found in 16/106 (15.09%) and in 15/106 (14.15%) of the blood and milk samples, respectively. In one case, a goat had a positive RT-PCR result in its milk, while no antibodies or DNA were detected in its corresponding serum and blood samples (Table 5).

Based on Log-linear statistical analysis as a version of chi-square analysis, the chi-square value, designated as G2, was equal to 38.96, and the variance between different proportions or rates of *T. gondii* infection using iELISA and RT-PCR assays in serum–blood–milk triplet samples was highly significant (*p* < 0.0001), which showed a substantial difference in the ability of the aforementioned tests for *T. gondii* detection in various biological samples.

### 3.6. Risk Factor Assessment for T. gondii Infection Using Individual Sera and Milk Samples

The seroprevalence in the female goats was 48.11%. It was higher in Terai Bainen (31/58; 53.45%) than in Tiberguent (6/14; 42.86%) and Zeghaia (14/34; 41.18%), but the difference was not statistically significant (*p* > 0.05) (Table 6). There was a non-significant difference in the prevalence of *T. gondii* in different age categories using both serological and molecular tools. The other variables considered were also not statistically significant.

Regarding the serological results, the goats tested in this study were more susceptible to *T. gondii* infection in autumn than in other seasons (72.73%; OR= 4.3077; *p* = 0.0354) (Table 6). No multivariate analysis was carried out, considering that only one variable appeared to be a risk factor.

The highest number of positive RT-PCR results was achieved in the second phase of lactation (6 out of 48 examined samples) and at the end of lactation (5 out of 17 examined samples). A less frequent detection of parasitic DNA was achieved in the first phase of lactation; 4/41 milk samples scored positive (Table 6). The comparison carried out regarding the prevalence of *T. gondii* DNA in serum and milk samples showed a variation according to immunization (IgG antibodies detection) and the lactation period, however, the difference was not significant (*p* > 0.05).

### 3.7. Genotyping

Only one among four samples (n° 194 from goat milk collected in the Tiberguent district) gave genotyping results. However, its genotyping could not be completed, and only one marker (B18) was amplified, giving a length of 158 bases. There were not enough amplified markers to conclude on a lineage for this strain, but we know that it is not the type I or type III lineage strain (because in lineage I and III, the B18 marker is 160). The genotypes of all positive controls (RH, ME49, and VEG) were confirmed (type I, type II, and type III, respectively).

## 4. Discussion

In the present study, we investigated *Toxoplasma gondii* infection via the detection of IgG antibodies in serum and the parasite’s DNA in blood and milk samples collected from naturally infected goats in familial herds in northeastern Algeria.

The goats in the study region were found to be highly exposed to *T. gondii*, with overall seroprevalence rates of 48.11% and 100% at individual and herd level, respectively. Serological screenings conducted on goat farms across different agro-ecological areas in Algeria revealed a *T. gondii* rate ranging from 11.9% to 78% [22,23,34,35].

The current seropositivity rate was higher than that reported in different countries, such as in China (29.54%) [36] and Pakistan (25.5%) [37]. However, higher prevalence rates of *T. gondii* were reported in Bulgaria (59.8%) [38] and Italy (60.6%) [11]. Comparable findings were documented in Romanian dairy goats (52.8%) [39]. The present high seropositivity rate suggests that goats are highly susceptible to *T. gondii* infection and more exposed to the parasite, which may be due to their access to contaminated pastures and water sources [23,39]. The variations in prevalence values among studies may be influenced by various factors, such as the density of infected cats shedding oocysts and contaminating the environment, breed susceptibility, feeding habits, rearing patterns, and climatic conditions [23,35,40].

*T. gondii* DNA was found in 15.09% of blood samples tested in the current study. Comparable results (13%) were recorded in a previous study from the far northeast of Algeria [41]. In Italy, 13% of blood and milk samples from goats were PCR-positive [11]. Discrepancies in these rates may be attributed to the variety of diagnostic methods used, husbandry systems, and wet conditions, which can impact oocysts’ survival [42].

For the first time, this survey revealed the presence of *T. gondii* DNA in the milk of naturally infected goats, since 15 out of 106 milk samples were PCR-positive. These results were higher than those reported in Poland (3%) [43] and Brazil (6%) [16] and comparable to those found in Slovakia (33%) by Spišák et al. [44] and in a recent Brazilian study (38.6%) [45]. However, a Tunisian study reported a lower prevalence (7.8%) of *T. gondii* DNA in goat milk, inconsistent with our results [20]. Conversely, Sroka et al. [46] reported a much higher *T. gondii* PCR-positive rate (65%) in Poland. Mohammadi Khamsian et al. [42] recently reported that 5.5% (11/200) of goat milk samples in Iran were infected with *T. gondii*.

The present results showed that only six seropositive goats showed positivity on PCR for both their blood and milk samples. The lower prevalence of *T. gondii* DNA detected by PCR in comparison to anti-*T. gondii* antibodies may have been because IgG antibodies are produced late in the infection, and the parasite is localized in the organs and tissues rather than circulated in the blood, finally reaching the milk [2,12]. Two goats with negative serology were found to have milk containing *T. gondii* DNA, which may have been due to the undetectable level of IgG-specific antibodies during the early stage of infection, as also suggested by Sroka et al. [46]. Furthermore, the antibody levels in sera may be affected by the phase of lactation [47]. The lactation phase did not appear to influence the excretion of *T. gondii* DNA in goat milk in the current study. In one case, a goat’s milk had a positive PCR result, but the corresponding serum and blood samples did not contain either antibodies or DNA. This animal may have been in the early stage of *T. gondii* infection, exhibiting an immune response following initial contact with the parasite (no reactivation), but had produced specific antibodies at a titer that was too low to be detected by serological testing.

Several studies have reported that only a small number of infected animals excrete parasite DNA in their milk [20,48]. *T. gondii* DNA was detected in 13 out of 51 seropositive goats (25.49%), which is higher than the detection rate reported in Italy, for example (10/77, 12.98%) [11]. Amairia et al. [20] found that only 2 out of 77 seropositive goats were releasing *T. gondii* DNA in their milk. The discordance in these results may be related to the phase of infection, stage of lactation, and immune status of the female goats at the time of sampling. Moreover, the occurrence of *T. gondii* DNA or tachyzoites has been reported in the milk of several animal species, including small ruminants [10,49,50]. The excretion of *T. gondii* in goat milk is irregular and dependent on the infection of milk-secreting cells. In experimentally infected goats, *T. gondii* was detected by PCR more consistently, but there was no correlation between the detection of viable *T. gondii* by bioassay in mice and *T. gondii* DNA by PCR, which indicates that *T. gondii* can be excreted in goat milk and can survive in fresh cheese made by cold-enzyme treatment [12]. The parasite stage excreted is the tachyzoite and/or tissue cysts containing bradyzoites [51,52]. The shedding of *T. gondii* tachyzoites in the milk of chronically infected goats is likely due to the revival of bradyzoites in the tissues during the peripartum period [49,53,54,55]. Moreover, despite the detection of parasitic DNA in raw goat milk, the shedding of viable parasites in milk appears to be low [45]. *T. gondii* excretion is more prevalent at the beginning and the end of lactation, particularly during the first two months [12,48,49]. Consistently, *T. gondii* DNA excretion was highly reported during the third phase of lactation. Steffen et al. [56] showed that pregnancy, kidding, and the peak of lactation are stressful physiological periods, facilitating the reactivation of chronic *T. gondii* infections, which are expressed by higher antibody titers. However, the molecular detection of *T. gondii* in goat milk seems to be the most effective direct method to detect the parasite in these samples, because, besides not requiring immediate processing, it can more accurately identify goats naturally exposed to this parasite [57].

The present serology results revealed that nearly all of the animals had a chronic infection, confirming that even healthy goats can release the parasite in their milk. According to Walsh et al. [58], tachyzoites can survive for three to seven days at + 4° C in milk, and they can reach the digestive tract without being destroyed, possibly causing an infection [13,59]. Thus, in the current study, the molecular detection of *T. gondii* DNA in milk could pose a potential risk for humans to be infected via the consumption of contaminated raw milk or milk-derived products. A similar insight has also been reported by other authors [60,61]. Nevertheless, the presence of DNA does not mean that alive tachyzoites are present in the biological sample [5].

However, the simultaneous presence of anti-*T. gondii* IgG antibodies and protozoan DNA in milk was observed in 25.49% (13/51) of the seropositive goats. Therefore, seronegative animals with the detection of *T. gondii* DNA in their milk (3.63%; 2/55) may have been in the early stage of infection, when their IgGs were not present in sufficient quantities to be detected by the serological test. Alternatively, these animals may have been recently infected with the parasite. In addition, no correlation has been reported between the results of PCR-based DNA detection and the serological status of lactating animals [62].

PCR results from blood samples can determine if a seropositive goat is releasing *T. gondii* DNA into its milk. The present investigation was prompted by the findings that the consumption of unpasteurized goat’s milk is a risk factor for acquired toxoplasmosis in pregnant women from Algeria [24,25]. Furthermore, milk from goats with positive serological and molecular results was used to produce cheeses, which might be a potential risk for acquiring *T. gondii* infection because of tachyzoites surviving in fresh cheese for up to 10 days [63].

In Algeria, previous studies have identified a considerable prevalence of *T. gondii* in both humans and domestic animals, including goats. However, the genetic diversity of strains circulating within the country remains relatively understudied. Consequently, isolating parasite DNA from livestock, including goats, and genotyping the strains using highly discriminatory markers [31] could offer insight into the strains currently circulating within Algeria.

To the best of our knowledge, the present study, along with two previous Tunisian and Egyptian ones [64,65], may be the only North African studies comprehensive enough to investigate the genetic characterization of *T. gondii* circulating in goat flocks. This is the first Algerian report of this nature. Previous Algerian studies involving *T. gondii* genotyping in human clinical samples [66] and tissues from domestic cats [67] confirmed the dominance of genotype II strains, without the occurrence of any other clonal or recombinant types.

According to the earliest studies on *T. gondii* genotyping, the parasite displays types I, II, and III as its three main clonal lineages, in addition to a smaller number of recombinant strains [68]. Using multi-locus markers, more recent studies have shown a greater degree of genetic variety [31,68]. Strains from the type II lineage, the most common lineage associated with chronic infection, have been significantly more common in both human and animal populations [69]. Type III, which is less frequently found, is mainly linked to infection in domestic animals, and has been occasionally identified [46].

Furthermore, several studies have demonstrated the predominance of lineage II throughout the Mediterranean region and beyond [48,68,70,71]. In Africa, this lineage was associated with abortion in small ruminants [72], and it was the predominant strain in the North African region, followed by type III strains, which have been identified in some countries such as Egypt and Tunisia [65,73,74], instead of type I strains, which have never been isolated previously [68,75].

The typing of *T. gondii* DNA obtained from goat milk is not well-documented and has never been performed in such samples from Algeria. The only isolate that could be partially identified most probably belongs to type II, but we cannot exclude an atypical type. Type II strains, some variant type II strains, and some haplogroup 16 or atypical strains have a B18 at 158 bases [68,76]. Consequently, the most likely hypothesis is this *T. gondii* strain belongs to type II.

The current results suggesting the presence of *T. gondii* strains in goat milk, whatever the genotype II, is alarming, as this represents a risk of human congenital toxoplasmosis [77]. Mancianti et al. [11] revealed that genotype I occurred in only one PCR-positive milk sample, while genotype III predominated. Only the type III lineage was previously determined in goat milk from Poland [46]. In a recent Egyptian study, using nested PCR-RFLP of the SAG2 locus, and in agreement with the current results, it was found that all the positive milk samples from female goats belonged to the type II lineage [74]. In contradiction, the genotyping of Egyptian *T. gondii* isolates from caprine blood revealed the presence of only the type III lineage based on the PCR-RFLP technique for the SAG2 locus [73]. A study carried out on meat from Tunisian sheep showed the circulation of *T. gondii* isolates belonging to the three clonal lineages [78], reflecting the genetic diversity of *T. gondii* strains in Tunisia compared to Algeria, where almost all *T. gondii* strains belong to the type II lineage [66,67].

However, in these studies, the use of a single or a few genetic markers for typing assays may result in biased genetic identification and the definitive analysis may be limited. *T. gondii* isolates previously classified as clonal type II or III may, in fact, exhibit type I or atypical genotypes [46]. To ensure accurate genotyping, in these studies, as well as in ours, it is necessary to study discriminating genetic markers in sufficient numbers [79], including the GRA6 gene marker, which has sufficient polymorphism to detect three types of *T. gondii* genotypes in various hosts [80].

Genetic characterization using 15 microsatellite markers from this study was unsuccessful, which might be attributed to the low amount of *T. gondii* in milk relating to the intermittent excretion of this parasite [9]. In a recent Tunisian study, strains associated with human congenital toxoplasmosis were determined to be of the type II lineage using MS genotyping [81].

The assessment of the possible clonal type II lineage in this study corresponds with the findings of previous studies, which demonstrated a more generalized predominance of the type II lineage in North Africa and other Mediterranean countries [65,68]. The hypothesized occurrence of this lineage in Algerian goats is expected, given that this lineage was previously identified in two previous studies on human and cat samples from central Algeria [66,67], which might reflect a limited genetic diversity in the population structure of *T. gondii*.

## 5. Conclusions

The prevalence of anti-*Toxoplasma gondii* antibodies in goat sera, as well as the detection of *T. gondii* DNA in goat blood and milk, suggest that goats are significantly exposed to this parasite. The analysis of the variation in the detection of specific IgG in serum and *T. gondii* DNA in milk provided information on the optimal period in which DNA detection in milk should be performed. The mechanism underlying these changes deserves further investigation. The presence of a positive PCR result and a negative serologic one directed more attention to the technical diagnosis of toxoplasmosis, especially in lactating goats, and indicates that serological methods should be used in conjunction with PCR for more accurate diagnoses of *T. gondii* infection in livestock. The data obtained endorsed the possibility of using milk in the screening of *T. gondii* infection on goat farms and indicated the potential role of raw goat milk and milk-derived products in human infection by this protozoan, especially when milk is widely consumed in natura as a food source for rural families.

To the best of our knowledge, the present survey is the first study aiming to describe the genetic characterization of *T. gondii* circulating in goat flocks in Algeria. The genetic characterization of DNA isolates from milk samples from this region suggested the occurrence of type II strains. The present results point out the necessary of increasing genotyping and sampling efforts to accurately estimate *T. gondii* diversity in Algeria with phylo-geographical analysis. Therefore, knowledge of the molecular prevalence of *T. gondii* in goats is of interest for designing control strategies for toxoplasmosis in goats and limiting food transmission to humans in the context of one health.

## Figures and Tables

**Table 1:** Prevalence and statistical analysis of *T. gondii* DNA detection in blood and milk of goats from northeastern Algeria.

		RT-PCR Milk			McNemar Test		Kappa Coefficient	
		Positive	Negative	Total	*p*	OR (95% CI)	K	SE (95% CI)
**RT-PCR Blood**	Positive	7	9	16 (15.09%)	1	1.125 (0.434–2.9159)	0.3578	0.1238 (0.1149–0.6006)
	Negative	8	82	90				
	Total	15 (14.15%)	91	106				

*p*—*p*-value; OR—Odd Ratio; K—Kappa value; SE—Standard Error; CI—Confidence Interval.

**Table 2:** Prevalence and statistical analysis of *T. gondii* DNA detection in blood and milk of seropositive goats from northeastern Algeria.

		RT-PCR Milk			McNemar Test		Kappa Coefficient	
		Positive	Negative	Total	*p*	OR (95% CI)	K	SE (95% CI)
**RT-PCR Blood**	Positive	6	9	15 (29.41%)	0.8036	1.2857 (0.4788–3.4523)	0.2139	0.1467 (0–0.5013)
	Negative	7	29	36				
	Total	13 (25.49%)	38	51				

*p*—*p*-value; OR—Odd Ratio; K—Kappa value; SE—Standard Error; CI—Confidence Interval.

**Table 3:** Prevalence and comparison of *T. gondii* detection using iELISA and RT-PCR in goat milk from northeastern Algeria.

		RT-PCR Milk			McNemar Test		Kappa Coefficient	
		Positive	Negative	Total	*p*	OR (95% CI)	K	SE (95% CI)
**iELISA Serum**	Positive	13	38	51 (48.11%)	<0.000001	19 (4.5837–78.7574)	0.2243	0.06895 (0.08914–0.35945)
	Negative	2	53	55				
	Total	15 (14.15%)	91	106				

*p*—*p*-value; OR—Odd Ratio; K—Kappa value; SE—Standard Error; CI—Confidence Interval.

**Table 4:** Prevalence and comparison of *T. gondii* detection using iELISA and RT-PCR in goat blood from northeastern Algeria.

		RT-PCR Blood			McNemar Test		Kappa Coefficient	
		Positive	Negative	Total	*p*	OR (95% CI)	K	SE (95% CI)
**iELISA Serum**	Positive	15	36	51 (48.11%)	<0.000001	36 (4.9356–262.5797)	0.28300	0.06974 (0.14630–0.41969)
	Negative	1	54	55				
	Total	16 (15.09%)	90	106				

*p*—*p*-value; OR—Odd Ratio; K—Kappa value; SE—Standard Error; CI—Confidence Interval.

**Table 5:** Log-linear statistical analysis of serological and molecular results of *T. gondii* detection in serum–blood–milk triplet samples from goats in northeastern Algeria.

		iELISA Serum +			iELISA Serum −			Log-Linear Analysis		
		RT-PCR Blood +	RT-PCR Blood −	Total	RT-PCR Blood +	RT-PCR Blood −	Total	G^2^	df	*p*
**RT-PCR Milk**	+	6	7	13	1	1	2	38.96	2	<0.0001
	−	9	29	38	0	53	53			
	Total	15	36	51	1	54	55			

*p*—*p*-value; G^2^—chi-square value; df—degree of freedom.

**Table 6:** Risk factor analysis for *T. gondii* occurrence in serum and milk samples of goats from northeastern Algeria.

Factor	Category	iELISA Serum Positive/Tested (%)	OR (95% CI)	*p*	RT-PCR Milk Positive/Tested (%)	OR (95% CI)	*p*
**Locality**	Zeghaia	14/34 (41.18)	Ref	0.4795	5/34 (14.70)	Ref	0.7189
	Terai Bainen	31/58 (53.45)	0.6097 (0.259–1.435)		9/58 (15.52)	0.9387 (0.2868–3.0725)	
	Tiberguent	6/14 (42.86)	1.5309 (0.4715–4.9699)		1/14 (7.14)	2.3878 (0.2769–20.5933)	
**Age**	˂2 years	9/22 (40.91)	Ref	0.4045	2/22 (9.09)	Ref	0.3734
	2–5 years	36/68 (52.94)	0.6154 (0.2323–1.6302)		12/68 (17.65)	0.4667 (0.096–2.2694)	
	>5 years	6/16 (37.50)	1.875 (0.6127–5.7384)		1/16 (6.25)	3.2143 (0.3865–26.7284)	
**Phase of Lactation**	1st (1–45 days)	17/41 (41.46)	Ref	0.1182	4/41 (9.76)	Ref	0.134
	2nd (46–90 days)	22/48 (45.83)	0.8371 (0.3608–1.9423)		6/48 (12.50)	0.7568 (0.1981–2.8905)	
	3rd (91–120 days)	12/17 (70.58)	0.3526 (0.1075–1.1563)		5/17 (29.41)	0.3429 (0.089–1.3214)	
**Management system**	Intensive	4/5 (80)	Ref	0.2049	1/5 (20)	Ref	0.2753
	S. extensive	9/24 (37.5)	6.6667 (0.6409–69.3466)		1/24 (4.17)	5.75 (0.2955–111.8848)	
	S. intensive	38/77 (49.35)	0.6158 (0.2407–1.5755)		13/77 (16.88)	0.214 (0.0265–1.7289)	
**Abortion history**	Not aborted	24/56 (42.86)	Ref	0.3304	6/56 (10.71)	Ref	0.40343
	Aborted	27/50 (54.00)	0.6389 (0.2965–1.3767)		9/50 (18.00)	0.5467 (0.1797–1.6629)	
**Presence of cats**	No	7/15 (46.67)	Ref	1	2/15 (13.33)	Ref	1
	Yes	44/91 (48.35)	0.9347 (0.3128–2.7926)		13/91 (14.29)	0.9231 (0.1863–4.5736)	
**Season**	Autumn	8/11 (72.73)	Ref	0.0354 *	2/11 (18.18)	Ref	0.9581
	Winter	13/34 (38.24)	4.3077 (0.9647–19.2361)		5/34 (14.71)	1.2889 (0.2126–7.8156)	
	Spring	22/51 (43.14)	0.816 (0.3363–1.9798)		7/51 (13.73)	1.0837 (0.3137–3.7439)	
	Summer	8/10 (80)	0.1897 (0.0366–0.9832)		1/10 (10)	1.4318 (0.1563–13.1125)	

*p*—*p*-value; OR—Odd Ratio; CI—Confidence Interval; Ref—Reference value; *—*p*-value < 0.05.

## Data Availability

The data presented in this study are available on request from the corresponding author.

## Supplementary Materials

The following supporting information can be downloaded at: https://www.mdpi.com/article/10.3390/pathogens14020174/s1. Table S1: Sampling information of the 106 female goats randomly selected in Mila province, Algeria.
