# Supplementary material for: First Molecular Detection of Toxoplasma gondii DNA in Blood and Milk of Goats from Algeria
Source: Pathogens. 2025 Feb 10;14(2):174. doi: 10.3390/pathogens14020174 (PMC11858173; doi:10.3390/pathogens14020174)
Supplement: Supplementary file 1 [file pathogens-14-00174-s001.zip › pathogens-3437102-supplementary.pdf]

Table S1. Sampling information of the 106 female goats randomly selected in Mila province, Algeria.

N° Sample	Age	Phase of Lactati	Husbandry practice	Presence of Cats	Hygiene	History of abortion	Province	Locality
1	A	Beginning	S.int	Yes	Good	NA	Mila	Zeghaia
2	C	Mi-Lact	S.int	Yes	Good	A	Mila	Zeghaia
3	A	Mi-Lact	S.int	Yes	Good	NA	Mila	Zeghaia
4	B	Beginning	S.int	Yes	Good	NA	Mila	Zeghaia
6	B	End	S.int	Yes	Good	A	Mila	Zeghaia
11	B	End	S.int	Yes	Good	NA	Mila	Zeghaia
13	B	End	S.int	No	Good	NA	Mila	Zeghaia
15	C	Mi-Lact	S.int	No	Good	NA	Mila	Zeghaia
16	B	Beginning	S.int	No	Good	A	Mila	Zeghaia
121	B	Beginning	Int	Yes	Good	A	Mila	Bainen
122	C	Beginning	Int	Yes	Good	NA	Mila	Bainen
124	B	End	Int	Yes	Good	A	Mila	Bainen
125	A	Mi-Lact	Int	Yes	Good	A	Mila	Bainen
126	A	End	Int	Yes	Good	NA	Mila	Bainen
127	B	Mi-Lact	S.ext	Yes	Bad	A	Mila	Bainen
128	C	Mi-Lact	S.ext	Yes	Bad	A	Mila	Bainen
135	B	End	S.int	Yes	Bad	A	Mila	Zeghaia
136	B	End	S.int	Yes	Bad	A	Mila	Zeghaia
147	A	Beginning	S.int	Yes	Good	A	Mila	Tiberguent
148	B	Mi-Lact	S.int	Yes	Good	A	Mila	Tiberguent
150	B	Mi-Lact	S.int	Yes	Good	A	Mila	Tiberguent
151	A	Mi-Lact	S.int	Yes	Good	NA	Mila	Tiberguent
152	B	Beginning	S.ext	No	Bad	NA	Mila	Zeghaia
153	B	Beginning	S.ext	No	Bad	NA	Mila	Zeghaia
157	A	Mi-Lact	S.int	Yes	Good	A	Mila	Zeghaia
158	B	Beginning	S.int	Yes	Good	NA	Mila	Zeghaia
159	A	End	S.int	Yes	Good	NA	Mila	Zeghaia
185	A	Beginning	S.ext	Yes	Good	NA	Mila	Tiberguent
187	B	Mi-Lact	S.ext	Yes	Good	A	Mila	Tiberguent
191	A	Mi-Lact	S.int	Yes	Good	NA	Mila	Tiberguent
192	B	Beginning	S.int	Yes	Good	A	Mila	Tiberguent
193	B	Mi-Lact	S.int	Yes	Good	A	Mila	Tiberguent
194	B	Mi-Lact	S.int	Yes	Good	A	Mila	Tiberguent
198	B	Mi-Lact	S.int	Yes	Good	A	Mila	Tiberguent
220	B	Mi-Lact	S.int	Yes	Bad	A	Mila	Bainen
222	A	Beginning	S.int	Yes	Bad	NA	Mila	Bainen
223	B	Mi-Lact	S.int	Yes	Bad	NA	Mila	Bainen
228	B	Beginning	S.int	Yes	Bad	A	Mila	Bainen
229	B	Beginning	S.int	Yes	Bad	NA	Mila	Bainen
298	A	Mi-Lact	S.ext	Yes	Good	A	Mila	Zeghaia
301	B	Beginning	S.ext	Yes	Good	A	Mila	Zeghaia
302	B	Beginning	S.ext	Yes	Good	A	Mila	Zeghaia
305	B	Mi-Lact	S.ext	Yes	Good	A	Mila	Zeghaia
306	B	End	S.ext	Yes	Good	NA	Mila	Zeghaia
307	A	Mi-Lact	S.int	Yes	Good	A	Mila	Bainen
308	B	Mi-Lact	S.int	Yes	Good	NA	Mila	Bainen

310	B	Beginning	S.int	Yes	Good	A	Mila	Bainen
311	B	Beginning	S.int	Yes	Medium	NA	Mila	Bainen
313	B	Mi-Lact	S.int	Yes	Medium	A	Mila	Bainen
314	A	Mi-Lact	S.int	Yes	Medium	A	Mila	Bainen
315	B	Beginning	S.int	Yes	Medium	A	Mila	Bainen
316	B	End	S.int	Yes	Medium	NA	Mila	Bainen
317	B	End	S.int	Yes	Medium	NA	Mila	Bainen
319	B	Mi-Lact	S.int	Yes	Good	NA	Mila	Bainen
320	B	Mi-Lact	S.int	Yes	Good	A	Mila	Bainen
327	C	Beginning	S.ext	Yes	Bad	NA	Mila	Tiberguent
328	B	Beginning	S.int	Yes	Good	A	Mila	Zeghaia
329	A	Beginning	S.int	Yes	Good	NA	Mila	Zeghaia
330	B	Beginning	S.int	Yes	Good	NA	Mila	Zeghaia
332	C	Mi-Lact	S.int	Yes	Good	A	Mila	Zeghaia
333	B	Mi-Lact	S.int	Yes	Good	NA	Mila	Zeghaia
335	A	Mi-Lact	S.ext	Yes	Bad	A	Mila	Tiberguent
336	B	Beginning	S.ext	Yes	Bad	NA	Mila	Tiberguent
342	B	Beginning	S.int	Yes	Bad	NA	Mila	Bainen
343	A	Mi-Lact	S.int	Yes	Bad	A	Mila	Bainen
344	B	Beginning	S.int	Yes	Bad	NA	Mila	Bainen
345	B	Mi-Lact	S.int	Yes	Bad	A	Mila	Bainen
346	B	Beginning	S.ext	Yes	Good	NA	Mila	Zeghaia
347	A	Mi-Lact	S.ext	Yes	Good	NA	Mila	Zeghaia
348	B	Mi-Lact	S.ext	Yes	Good	A	Mila	Zeghaia
355	C	Beginning	S.ext	No	Good	A	Mila	Bainen
356	B	Mi-Lact	S.ext	No	Good	NA	Mila	Bainen
357	B	End	S.ext	No	Good	NA	Mila	Bainen
358	A	End	S.ext	No	Good	A	Mila	Bainen
359	A	End	S.ext	No	Good	NA	Mila	Bainen
570	B	Mi-Lact	S.int	Yes	Medium	NA	Mila	Zeghaia
571	B	Mi-Lact	S.int	Yes	Medium	A	Mila	Zeghaia
572	B	Beginning	S.int	Yes	Medium	NA	Mila	Zeghaia
576	B	Mi-Lact	S.int	Yes	Good	NA	Mila	Zeghaia
577	B	Mi-Lact	S.int	Yes	Good	NA	Mila	Zeghaia
581	B	Beginning	S.int	Yes	Medium	NA	Mila	Bainen
582	C	Mi-Lact	S.int	Yes	Medium	A	Mila	Bainen
583	C	Mi-Lact	S.int	Yes	Medium	NA	Mila	Bainen
584	B	End	S.int	Yes	Medium	A	Mila	Bainen
585	B	Beginning	S.int	Yes	Medium	NA	Mila	Bainen
586	C	Beginning	S.int	Yes	Medium	A	Mila	Bainen
587	B	Beginning	S.ext	No	Good	NA	Mila	Bainen
589	B	Mi-Lact	S.ext	No	Good	NA	Mila	Bainen
592	B	Mi-Lact	S.int	No	Good	NA	Mila	Bainen
594	C	Beginning	S.int	No	Good	NA	Mila	Bainen
596	A	Beginning	S.int	No	Good	NA	Mila	Bainen
605	B	Beginning	S.int	Yes	Good	NA	Mila	Bainen
608	A	Beginning	S.int	Yes	Good	NA	Mila	Bainen
609	B	Mi-Lact	S.int	Yes	Good	A	Mila	Bainen
610	B	Mi-Lact	S.int	Yes	Good	NA	Mila	Bainen
611	C	Mi-Lact	S.int	Yes	Good	NA	Mila	Bainen

612	C	Beginning	S.int	Yes	Good	A	Mila	Bainen
613	C	End	S.int	Yes	Good	A	Mila	Bainen
614	B	End	S.int	Yes	Good	NA	Mila	Bainen
615	B	Beginning	S.int	Yes	Good	A	Mila	Bainen
616	B	Mi-Lact	S.int	Yes	Good	A	Mila	Bainen
617	B	Beginning	S.int	Yes	Good	NA	Mila	Bainen
618	B	Mi-Lact	S.int	Yes	Good	A	Mila	Bainen
619	B	Mi-Lact	S.int	Yes	Good	NA	Mila	Bainen
620	C	Mi-Lact	S.int	Yes	Good	NA	Mila	Bainen
621	C	Beginning	S.int	Yes	Good	A	Mila	Bainen

A – Abortion; NA - No Abortion

A- animals under 2 years of age; B - animals are 2-5 years old C - animals over 5 years of age

Int- Intensive; S.int; Semi-intensive; S.ext – Semi-extensive

Season	PCR-Blood	PCR-Milk	Ab-Serum
Spring	0	0	0
Spring	0	0	0
Spring	0	0	0
Spring	0	0	0
Spring	0	0	1
Spring	0	0	1
Winter	0	1	1
Winter	0	0	1
Winter	0	0	1
Spring	0	0	1
Spring	0	0	0
Spring	1	0	1
Spring	1	1	1
Spring	0	0	1
Winter	0	0	1
Winter	0	0	0
Spring	1	1	1
Spring	0	0	1
Spring	0	0	1
Spring	0	0	0
Spring	0	0	0
Spring	0	0	1
Winter	0	0	0
Winter	0	0	0
Spring	0	0	0
Spring	0	0	1
Spring	0	0	0
Winter	0	0	0
Winter	0	0	0
Spring	0	0	1
Spring	0	0	0
Spring	0	1	1
Spring	0	1	1
Spring	0	0	1
Summer	0	0	1
Summer	0	0	1
Summer	0	0	1
Summer	0	0	1
Summer	0	0	0
Summer	0	0	0
Summer	0	1	1
Summer	0	0	1
Summer	1	0	1
Summer	1	0	1
Autumn	0	0	1
Autumn	0	0	1

Autumn	0	0	1
Winter	0	0	1
Winter	0	0	1
Winter	0	1	1
Winter	1	0	1
Winter	1	1	1
Winter	0	1	1
Spring	0	0	0
Spring	0	0	1
Winter	0	0	0
Spring	0	0	0
Spring	0	0	0
Spring	0	0	0
Spring	0	0	0
Spring	0	0	0
Winter	0	0	0
Winter	0	0	0
Winter	0	0	0
Winter	0	0	0
Winter	0	0	0
Winter	0	0	0
Spring	0	0	0
Spring	1	0	1
Spring	0	0	0
Spring	0	0	1
Spring	0	0	1
Spring	0	0	0
Spring	0	0	0
Spring	0	0	0
Spring	1	1	1
Spring	0	0	0
Spring	0	0	0
Spring	0	0	0
Spring	0	0	0
Autumn	0	0	0
Autumn	0	0	0
Autumn	0	0	1
Autumn	1	1	1
Autumn	1	0	1
Autumn	1	1	1
Autumn	0	0	0
Autumn	0	0	1
Winter	0	0	0
Winter	1	0	1
Winter	0	0	0
Winter	1	0	1
Winter	0	0	0
Winter	0	0	0
Winter	0	0	0
Winter	0	0	0

Winter	0	0	0
Winter	0	0	0
Winter	0	0	1
Winter	0	0	0
Spring	0	0	0
Spring	0	1	0
Spring	1	1	0
Spring	0	1	1
Spring	0	0	0
Spring	1	0	1
